# Unraveling the interplay of ferroptosis and immune dysregulation in diabetic kidney disease: a comprehensive molecular analysis

**DOI:** 10.1186/s13098-024-01316-w

**Published:** 2024-04-20

**Authors:** Yuanyuan Jiao, Xinze Liu, Jingxuan Shi, Jiaqi An, Tianyu Yu, Guming Zou, Wenge Li, Li Zhuo

**Affiliations:** 1 Department of Nephrology, Fuwai Hospital, National Center for Cardiovascular Diseases, Chinese Academy of Medical Sciences & Peking Union Medical College, 100037 Beijing, China; 2 Department of Nephrology, China-Japan Friendship Hospital, 100029 Beijing, China; 3 China-Japan Friendship Clinic Medical College, Beijing University of Chinese Medicine, 100029 Beijing, China; 4 Institute of Clinical Medical Sciences, China-Japan Friendship Hospital, 100029 Beijing, China; 5 China-Japan Friendship Clinic Medical College, Peking University, 100191 Beijing, China; 6 Department of Nephrology, China-Japan Friendship Hospital, Beijing, China No.2, East Yinghuayuan Street, 100029

**Keywords:** Diabetic kidney disease, Ferroptosis, Artificial intelligence, Immune landscape

## Abstract

**Background:**

Diabetic kidney disease (DKD) is a primary microvascular complication of diabetes with limited therapeutic effects. Delving into the pathogenic mechanisms of DKD and identifying new therapeutic targets is crucial. Emerging studies reveal the implication of ferroptosis and immune dysregulation in the pathogenesis of DKD, however, the precise relationship between them remains not fully elucidated. Investigating their interplay is pivotal to unraveling the pathogenesis of diabetic kidney disease, offering insights crucial for targeted interventions and improved patient outcomes.

**Methods:**

Integrated analysis, Consensus clustering, Machine learning including Generalized Linear Models (GLM), RandomForest (RF), Support Vector Machine (SVM) and Extreme Gradient Boosting (xGB), Artificial neural network (ANN) methods of DKD glomerular mRNA sequencing were performed to screen DKD-related ferroptosis genes.CIBERSORT, ESTIMATE and ssGSEA algorithm were used to assess the infiltration of immune cells between DKD and control groups and in two distinct ferroptosis phenotypes. The ferroptosis hub genes were verified in patients with DKD and in the db/db spontaneous type 2 diabetes mouse model via immunohistochemical and Western blotting analyses in mouse podocyte MPC5 and mesangial SV40-MES-13 cells under high-glucose (HG) conditions.

**Results:**

We obtained 16 differentially expressed ferroptosis related genes and patients with DKD were clustered into two subgroups by consensus clustering. Five ferroptosis genes (DUSP1,ZFP36,PDK4,CD44 and RGS4) were identified to construct a diagnostic model with a good diagnosis performance in external validation. Analysis of immune infiltration revealed immune heterogeneity between DKD patients and controls.Moreover, a notable differentiation in immune landscape, comprised of Immune cells, ESTIMATE Score, Immune Score and Stromal Score was observed between two FRG clusters. GSVA analysis indicated that autophagy, apoptosis and complement activation can participate in the regulation of ferroptosis phenotypes. Experiment results showed that ZFP36 was significantly overexpressed in both tissue and cells while CD44 was on the contrary.Meanwhile,spearman analysis showed both ZFP36 and CD44 has a strong correlation with different immune cells,especially macrophage.

**Conclusion:**

The regulation of the immune landscape in DKD is significantly influenced by the focal point on ferroptosis. Newly identified ferroptosis markers, CD44 and ZFP36, are poised to play essential roles through their interactions with macrophages, adding substantial value to this regulatory landscape.

**Supplementary Information:**

The online version contains supplementary material available at 10.1186/s13098-024-01316-w.

## Introduction

Diabetic kidney disease (DKD) is one of the most serious microvascular diseases in patients with diabetes and the main cause of end-stage renal disease (ESRD) [1]. The incidences of diabetes mellitus (DM) and DKD are increasing rapidly, and place a great burden on the healthcare system in China [1]. According to the 10th edition of the Global Diabetes Map, as of 2021, there were 537 million adult diabetic patients around the world, 140.9 million of whom are in China [2]. DKD has a prevalence of 20–40% in patients with diabetes, and eventually progresses to chronic renal failure [3]. Conventional therapeutic regimens such as glycemic control and the use of drugs such as Sodium-glucose Linked Transporter-2 (SGLT-2) inhibitors and Renin-Angiotensin-Aldosterone System (RAAS) inhibitors [3], although widely used, have limited therapeutic efficacy in the treatment of DKD, especially in patients with glomerular filtration rates less than 20 ml/min. Hence, a better understanding of its molecular mechanisms and molecular changes involved in DKD and the identification of novel molecular targets for DKD therapy are urgently needed.

Iron is an essential nutrient element and involved in many physiological processes including cell growth, metabolism, proliferation, and differentiation.Homeostasis of iron metabolism is modulated by systemic transport,average absorption and cellular storage and regulation,more and more evidence proved that the association between iron status and kidney diseases is inseparable, but iron overload in the body promotes cell death caused by membrane lipid peroxidation, namely ferroptosis [4–6].Since ferroptosis was first discovered in 2012,as a potential therapeutic way for various disease treatment has received generative concern [7, 8].Previous studies have shown that ferroptosis plays a vital role in various malignancies and has a strong correlation with tumor immune reactions [9, 10].Recently, ferroptosis has garnered enormous interest in non-neoplastic diseases,such as degenerative pathologies and numerous organ injuries [7].In addition to the diseases mentioned above, ferroptosis also plays a important role in DKD. Zhang et al.found that rosiglitazone, an inhibitor of acyl-CoA synthetase long chain family 4 (ACSL4), improved the renal function and decreased lipid peroxidation products and desensitised ferroptosis in DKD mice [11].Another research proved that the upregulation of Nrf2 by fenofibrate treatment suppresses diabetes-associated ferroptosis, thereby delaying the progression of DKD [12].Sp1-mediated upregulation of Prdx6 expression prevents podocyte injury in DKD via mitigation of oxidative stress and ferroptosis [13].The above suggested that to further study the role of ferroptosis in the diagnosis and treatment of DKD is a promising strategy.

The immune landscape mainly refers to immune cells and immune-related molecules in the immune microenvironment (IME). Previous studies have found that the tumor immune microenvironment plays a crucial role in regulating iron metabolism and homeostasis [14]. Many immune cells, such as innate immune cells (macrophages and neutrophils) and adaptive immune cells (T and B lymphocytes), are affected by ferroptosis [15]. Furthermore, ferroptosis has been found to synergize with immunomodulation, further impacting the immune landscape in different cancers [16]. However, the relationship between ferroptosis and the immune landscape in DKD has not been clearly expounded.

**Fig. 1:**
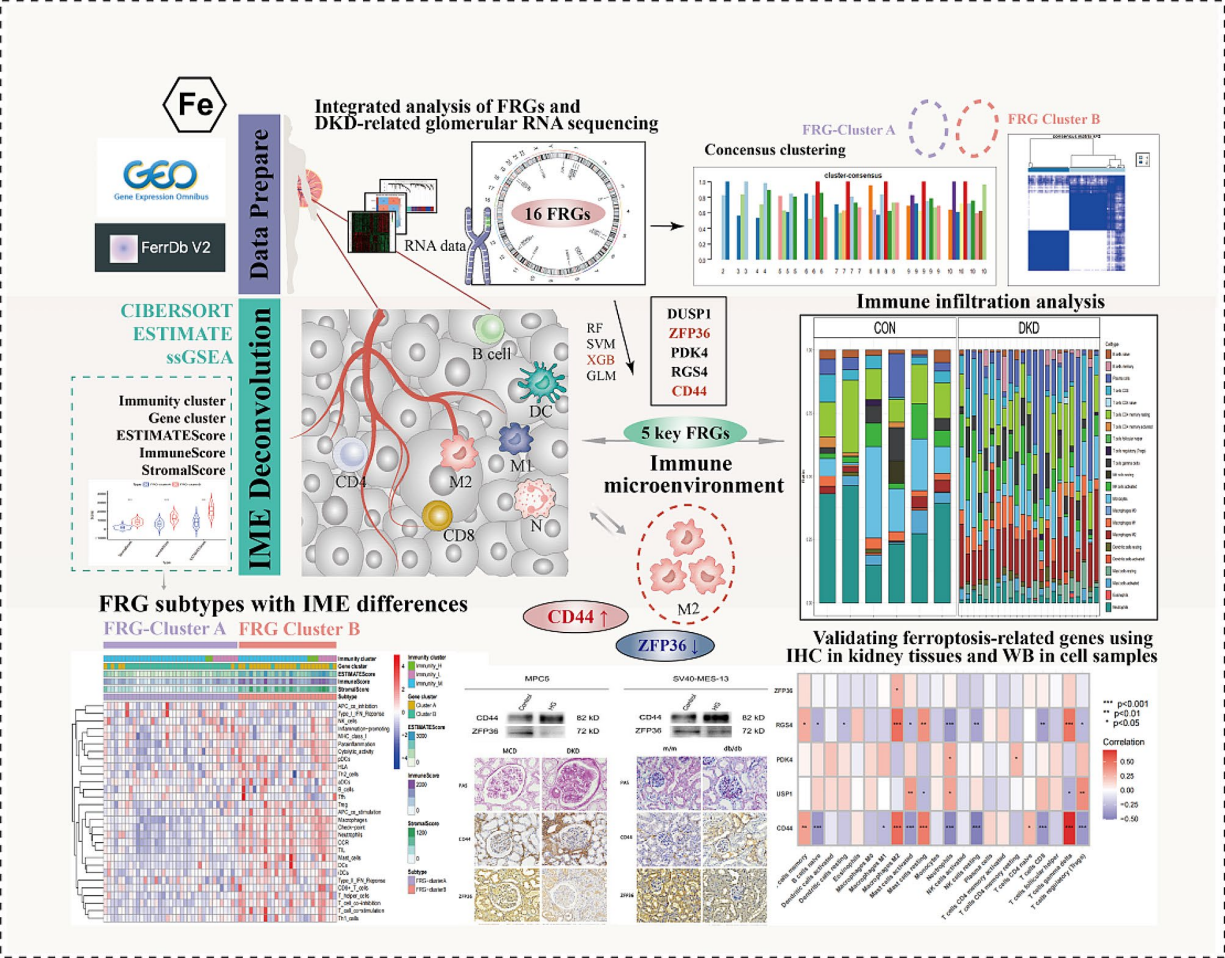
The flowchart of the study. FRG: Ferroptosis related genes, DKD: Diabetic kidney disease, CON: Control, IME:Immune microenvironment

In this study, we systematically assess the role of ferroptosis in the diagnosis of diabetic kidney disease and its relationship with the immune landscape. Initially, the diagnostic model is established by screening ferroptosis-related genes (FRGs) through artificial intelligence methods such as machine learning and artificial neural networks, benefiting the diagnosis of DKD and optimization of treatment strategies. Subsequently, based on the FRGs, the relationship between FRG-clustered subgroups, key genes, immune cells, and immune microenvironment (IME) scores is analyzed. Finally, our findings may help explore the mechanisms of how FRGs regulate the IME to identify potential therapeutic targets for the treatment of DKD. The specific details of the study are depicted in Fig. 1.

## Materials and methods

### Data collection

All datasets (GSE30528 [17], GSE104948 [18], GSE96804 [19],GSE47183 [20] and GSE142025 [21]) acquired from kidney transcriptional profiles of DKD patients and control group patients were downloaded from the NCBI-GEO database (http://www.ncbi.nlm.nih.gov/geo/) (Table 1). GSE30528, GSE104948, GSE96804 and GSE47183 were merged into a new matrix using the ComBat algorithm in the SVA R package in Bioconductor for batch correction of datasets to eliminate batch effects; this ultimately yielded a new matrix. Then we use the limma package for differentially expressed genes (DEGs) analysis with| logFC (fold change)| > 0.5 and adj.P.Val < 0.05 selected as the thresholds for screening. In addition, the volcano map and the heat map were visualized by the R software, which displayed the DEGs between two groups.

**Table 1:** Five datasets downloaded from the GEO database included in the study

Reference	GEO	Platform	Con	DKD
Woroniecka KI et al.	GSE30528	GPL571	13	9
Grayson PC et al.	GSE104948	GPL22945	18	7
Pan Y et al.	GSE96804	GPL17586	20	41
Ju W et al.	GSE47183	GPL14663	8	7
Fan Y et al.	GSE142025	GPL20301	9	27

### Weighted gene co-expression network analysis

The WGCNA R package was used to construct a weighted coexpression network of gene clusters most relevant to DKD. To ensure the reliability of the weighted coexpression network, the samples were normalised and outliers were removed. The soft threshold was determined by calculating the best value of the weighting parameter of the neighbor function based on pick soft threshold. Then the adjacency matrix was transformed into a topological overlap matrix (TOM), the corresponding phase dissimilarity measure (1 − TOM) was calculated, and the DKD onset-related gene modules were constructed via hierarchical clustering using a dynamic tree cutting method.

### Functional enrichment analyses of ferroptosis related genes

Ferroptosis-related genes (FRGs) were derived from the FerrDb V2 database (http://www.zhounan.org/ferrdb/) [22].The differentially expressed FRGs were obtained by intersecting the genes obtained using Limma,WGCNA and the FRGs with the VennDiagram R package.Then,the circos package were used to show the position of differentially expressed FRGs (DE-FRGs) on the chromosome map. Finally,the ggplot2 and cluster Profiler R packages were applied to conduct Gene Ontology (GO) and Kyoto Encyclopedia of Genes and Genomes (KEGG) pathway functional enrichment analyses, the results of which were drawn using the ggplot2 and enrichplot packages. Biological processes and signalling pathways were screened according to *p* < 0.05 and all results were visualised using the ggplot2 R package.

### Diagnosis of key DE-FRGs via GLM, RF, SVM, xGB and ANN

To discern the DE-FRGs with optimal diagnostic accuracy for predicting DKD, a comparative analysis of multiple artificial intelligence methodologies, namely the Generalized Linear Model (GLM), RandomForest (RF), Support Vector Machine (SVM), and Extreme Gradient Boosting (xGB), was undertaken within the caret R package. The selection of the most effective predictive method involved a meticulous evaluation of residual distribution and feature importance through the utilization of the modeling function within the Dalex R package. GLM extends the capabilities of the multiple linear regression model by flexibly estimating the relationship between features that exhibit correlation under a normal distribution, and both categorical and continuous independent features [23]. RF is a machine learning methodology that utilizes multiple standalone decision trees for the purpose of classification or regression predictions [24].SVM algorithm constructs a hyperplane in the feature space, aiming to achieve the maximum margin for effectively distinguishing positive outcomes from negative examples [25]. xGBoost is an ensemble of boosted trees based on gradient boosting, providing a nuanced balance between classification accuracy and model complexity during comparisons [26].Therefore, the optimal machine learning model was chosen, and the identification of the five most crucial variables was conducted to determine the primary predictor gene linked to DKD.Besides, a DKD diagnosis nomogram was constructed based on the key genes using the rms R package. Artificial neural network (ANN) model was constructed based on neuralnet package and predictive diagnostic value was exhibited by evaluating the area under the operating curve (AUC) in another DKD dataset (GSE142025) including 9control subjects and 27 patients with DKD. Before initiating ANN, it is imperative to standardize the maximum and minimum data values, and establish the number of hidden layers as 5. In the realm of parameter selection, there lacks a steadfast rule governing the fixed quantity of layers and neurons. Ideally, the number of neurons should be positioned between the input layer size and the output layer size, conventionally around two-thirds of the input size.We performed receiver operating characteristic (ROC) analysis using the pROC R package. DE-FRGs have an accurate diagnostic value in predicting DKD when AUC ≥ 0.7.

### Unsupervised clustering for 16 key DE-FRGs

Unsupervised clustering analysis was used to identify distinct ferroptosis modification patterns according to the expression of 16 FRGs and divide patients into different groups for further analysis. The number of FRG clusters and the stability of clusters were decided by the consensus clustering algorithm [27]. The R package “ConsensusClusterPlus” was applied to conduct the above steps for 1000 times to guarantee the stability of classifcation [28].Principal component analysis (PCA) was applied to test the effect of key DE-FRGs classification result.

### Comparison and estimation of immune cell infiltration

CIBERSORT, as a tool based on the principle of linear support vector regression, is widely used to assess immune cell types in the microenvironment [29]. It contains 547 biomarkers and defines 22 human immune cell phenotypes, covering plasma cells, B-cells, T-cells, and myeloid cell subpopulations. Immune cell gene expression profiles were downloaded from the CIBERSORT website, and the CIBERSORT R package was used to quantify immune cell infiltration. The obtained immune cell infiltration matrix was filtered at P < 0.05, and the results were visualized using the ‘corrplot’ R package. Moreover, we also used the ssGSEA (single-sample gene set enrichment analysis) method based on the immune gene set from a published article to assess the immune cell infiltration of each sample [30].

### Immune microenvironment comparison between ferroptosis regulation patterns

The ESTIMATE method was conducted to evaluate stromal score, immune score and ESTIMATE score. the ssGSEA algorithm was used to comprehensively estimate the immunological characteristics of each sample in this study based on 29 immune gene sets [31].We implement this analysis using estimate, GSVA, GSEABase, pheatmap and limma R packages.Moreover,the IME scores between different ferroptosis clusters were evaluated by Wilcoxon rank sum test.

### Gene set variation analysis

Gene set variation analysis (GSVA) method was applied to assess biological activities of the ferroptosis subtypes. The gene set documents “c2.cp.kegg.symbols” and “c5.go.symbols” used for running GSVA analysis were downloaded from the MSigDB database.

### Experimental materials

The study received approval from the Human Ethics Review Committee of the China-Japan Friendship Hospital (Approval Number: 2018-45-K34). All volunteers provided their informed consent and signed the necessary documents. All animal experiments were conducted in compliance with the ARRIVE guidelines and adhered to the regulations outlined in the U.K. Animals (Scientific Procedures) Act of 1986.The clinical samples were from DKD patients by renal biopsy (according to the relevant standards in the 2021 version of the Chinese DKD clinical diagnosis and treatment guidelines). The control group consisted of kidney tissues from 5 patients diagnosed with minimal change disease (MCD), whereas the experimental group comprised kidney tissues from 5 patients with DKD.The experimental mice were purchased from Beijing Charles River Laboratories (CRL) Experimental Animal Technology Co., Ltd., including 20 week old male SPF db/db spontaneous type 2 diabetes mice and their litter born m/m mice. The control group included 3 m/m mices with a normal genetic background while the experimental group comprised 3 db/db spontaneous type 2 diabetes mices.The mouse immortalized kidney podocyte line MPC5 cells were purchased from ATCC, and the mouse glomerular mesangial cell line SV40-MES-13 cells were purchased from Shanghai Fuheng Cell Bank.

Antibody for CD44 and Nephrin were purchased from Abcam (Cambridge, MA, UK), antibody for ZFP36 was purchased from Abcepta (Suzhou,China) and antibodies for GPX4 and TFR1 were from Affinity Biosciences (Cincinnati, OH, USA), and antibodies for Podocin, Ki67 and GAPDH were from Proteintech (Rosemont, IL, USA).ECL luminous solution was purchased from Beijing Dingguo Changsheng Biotechnology Co, Ltd.

### Immunohistochemical (IHC) staining

All specimens were fixed with 4% paraformaldehyde, embedded in paraffin, and cut into sections 4 μm   thick. The sections were rehydrated through a graded ethanol series, incubated in citrate buffer at 95 °C for 15 min for antigen retrieval, inactivated with H2O2 at room temperature for 10 min, penetrated with 0.1% Triton X-100 at room temperature for 10 min, and sealed with 5% BSA sealing solution at 37 °C for 1 h. Then, primary antibody to CD44, ZFP36, GPX4 or TFR1 was added dropwise and incubated at 37 °C for 2 h. After adding the corresponding secondary antibody and incubating the samples at room temperature for 20 min, 3,3′-diaminobenzidine (DAB) as a chromogen was added for 5–10 min. The reaction was terminated by adding H2O2 and the nuclei were stained with hematoxylin for 1 min. Then the sections were dehydrated through a graded ethanol series, made transparent with xylene, sealed with neutral gum, and observed and photographed under a microscope.

### Cell culture and grouping

MPC5 cells were treated with 10% FBS, 1% penicillin-streptomycin, and 10 U/mL γ-interferon, and then cultured in high-sugar DMEM. Cells at 70–80% confluence were collected to remove γ-interferon. MPC5 cells were further cultured and induced to differentiate for 1–2 weeks. The differentiated and mature MPC5 cells were used in subsequent experiments. SV40-MES-13 cells were cultured in DMEM/F12 medium containing 10% FBS and 1% penicillin-streptomycin. The cells were cultured in a 5% CO2 incubator at 37 °C for standby.MPC5 and SV40-MES-13 cells were divided into two groups. In a HERAEUS instrument, cells were cultured with 5.5 mmol glucose and 19.5 mmol mannitol as controls (Control), or with 25 mmol glucose as the high-glucose model group (HG).

### Western blotting

Cells from each group were incubated on ice in RIPA lysis buffer containing 1% PMSF for lysis to extract total protein. The protein concentration was determined using a BCA kit. Aliquots of 30 µg total protein were separated by 6–16% SDS-PAGE and transferred to membranes, which were then blocked with 5% skim milk, probed with primary antibody (CD44, ZFP36, GPX4, TFR1, Nephrin, Podocin, Ki67, and GADPH) overnight at 4 °C and incubated with horseradish peroxidase-conjugated secondary antibody at room temperature for 2 h. ECL luminous solution was used for colour development and images were taken.

### Statistical analysis

Statistical analyses were performed using R software (version4.3.0). Differences between two groups were examined for significance by Kruskal-Wallis test or Wilcoxon test. In all analyses, *p* < 0.05 was taken to indicate statistical significance.

## Results

### Acquisition of potential ferroptosis related genes of DKD

**Fig. 2:**
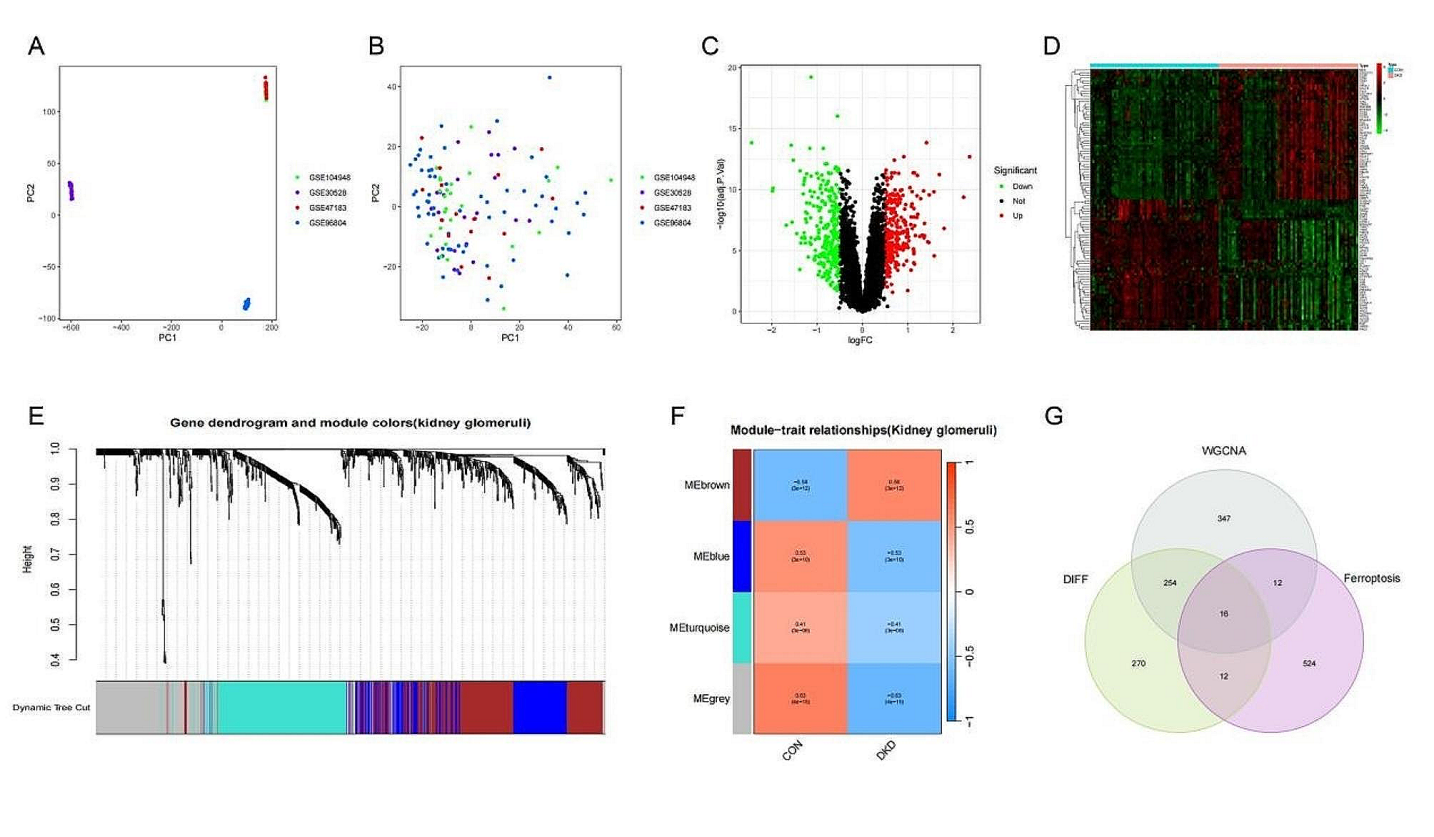
**(A) and (B)** The PCA before or after removing batch.**(C)**The volcano map of the DEGs.**(D)**The heatmap of the top 50 upregulated and top 50 downregulated DEGs.(**E and F**) Four modules construction and Module-trait relationship from WGCNA. **(G)**The Venn map of WGCNA,DIFF and ferroptosis. DIFF:differently expressed genes; WGCNA:weighted gene co-expression network analysis.

To identify potential ferroptosis related genes of DKD, we first integrated four datasets from GEO database and formed a new combined dataset including 59 control and 64 DKD human glomeruli samples.The PCA (Fig. 2A and B) analysis showed that the batch effect between raw data and merged data was better eliminated. Subsequently,we screened for the deviantly expressed genes and identified 552 DEGs that likely associated with the onset of DKD.The result was presented through a volcano plot (Fig. 2C), and depicted the top 50 upregulated and the top 50 downregulated DEGs in a heatmap (Fig. 2D). Through WGCNA analysis, four modules were constructed (Fig. 2E) and Module-trait relationship analysis showed that the grey module (0.63 (*P* = 4e − 15)) had the most significant positive correlation with the pathogenesis of DKD,followed by brown module(0.58 (*P* = 3e − 12)) (Fig. 2F),the grey module containing 286 genes and the brown module containing 343 genes, totally 629 genes were regarded as the key genes related to DKD from WGCNA method.After taking intersection of the two methods with 564 ferroptosis genes from FerrDb V2 database,a total of 16 DE-FRGs were finaly obtained (Fig. 2G).

### GO and KEGG enrichment analysis of DE-FRGs

**Fig. 3:**
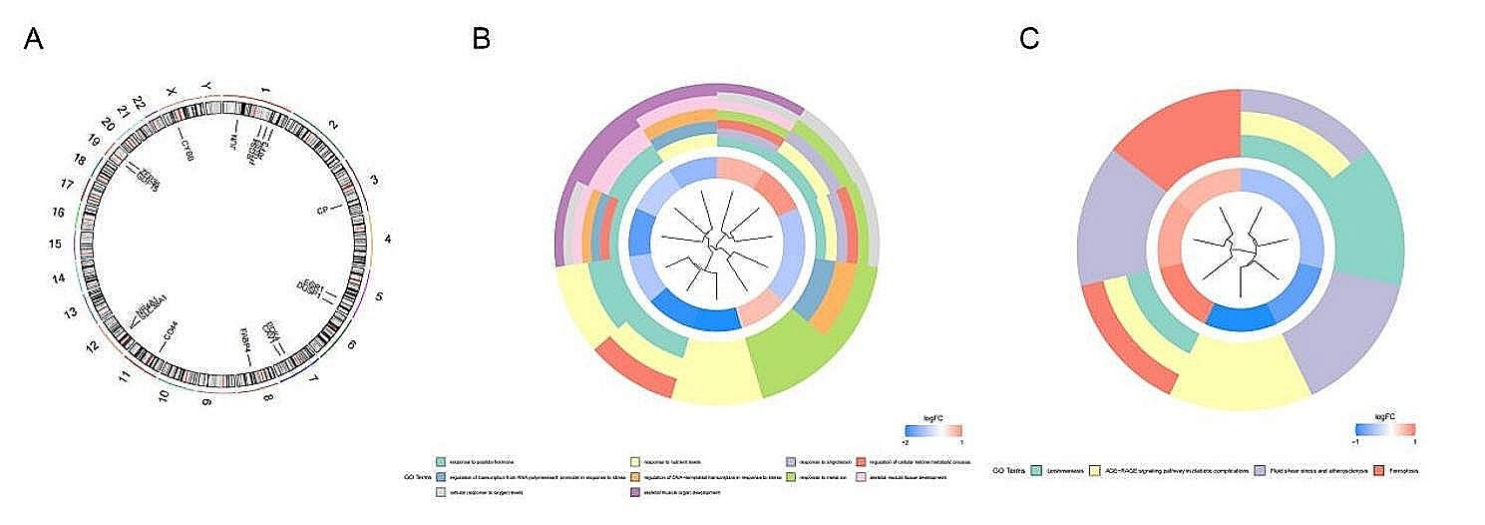
**(A)**The Chromosomal location of DE-FRGs. **(B)** GO annotation and **(C)** KEGG pathway enrichment analysis of DE-FRGs.

The location of DE-FRGs on chromosomes was displayed in Fig. 3A.GO analysis showed that DE-FRGs were mainly enriched in response to peptide hormone,response to nutrient levels, response to metal ion and etc. (Fig. 3B). KEGG pathway enrichment analysis of DE-FRGs found that the main enriched pathways were Leishmaniasis,ferroptosis and etc. (Fig. 3C).

### Detection and validation for hub DE-FRGs

**Fig. 4:**
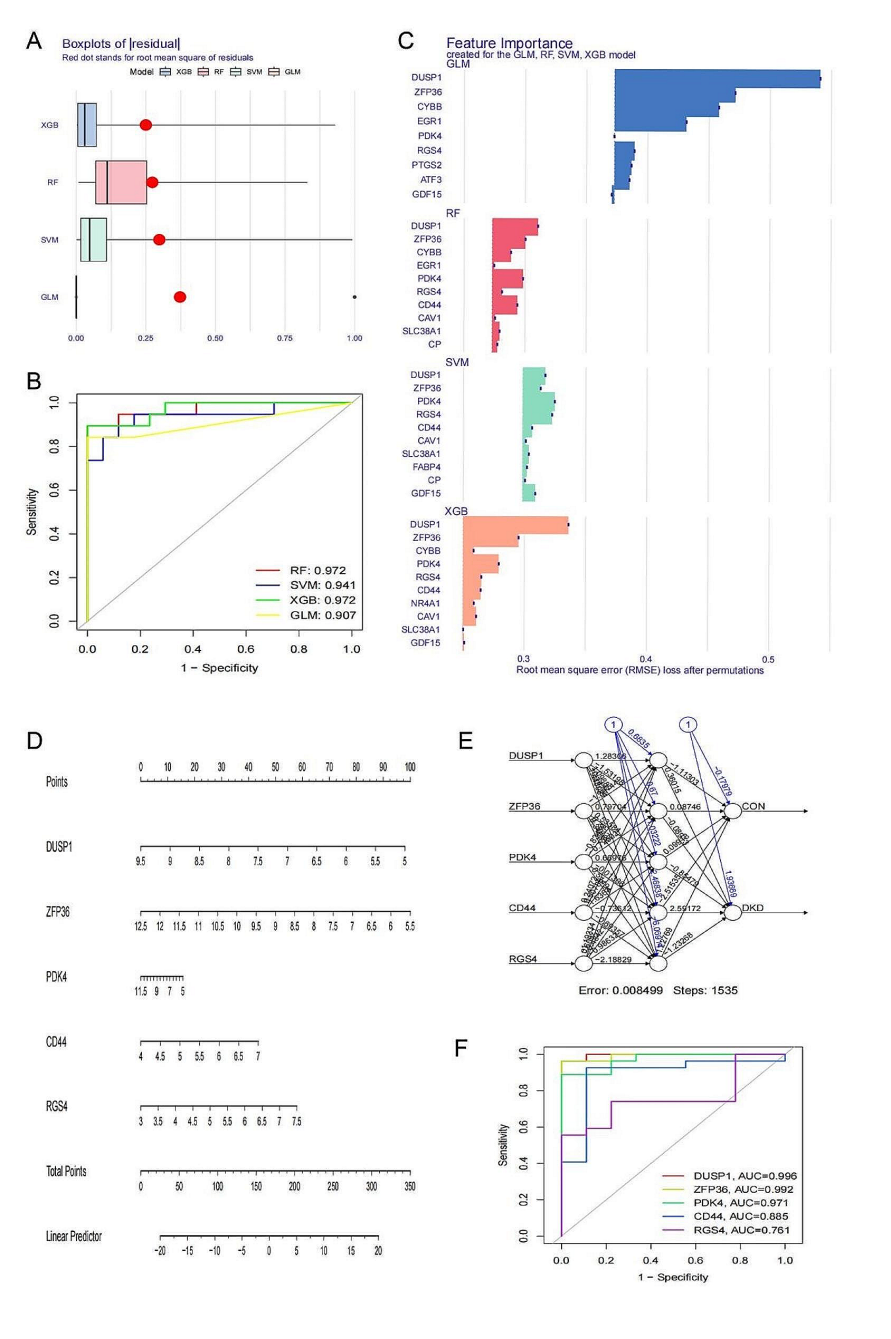
**(A)** The root mean square of residuals,**(B)** feature importance and **(C)** four machine learning methods; **(D)** Nomogram for diagnosing DKD;**(E)** ANN diagnostic model and **(F)** ROC analysis for vailidation.

The 16 DE-FRGs were put into four machine learning methods including RF, GLM, SVM and xGB. The xGB machine learning models resulted in the smallest residuals [Fig. 4A].Subsequently, the significant feature variables of each model’s top 10 were ordered based on root mean square error (RMSE) [Fig. 4C].Moreover, the discriminative efficacy of the four machine learning algorithms was assessed on the test set through the computation of receiver operating characteristic (ROC) curves using a fivefold cross-validation approach. The machine learning model exhibiting the greatest area under the curve (AUC) was chosen. Notably, the RF and xGB machine learning model demonstrated the highest AUC (AUC = 0.972, as depicted in Fig. 4B), indicating their superior performance in diagnosing Diabetic Kidney Disease (DKD).In summary, xGB machine learning model performed the best model in terms of root mean square of residuals and diagnostic performance compared with other three models [Fig. 4A-C].The five most important genes DUSP1,ZFP36,PDK4,CD44 and RGS4 from xGB model were selected as hub DE-FRGs.A nomogram for diagnosing DKD was constructed based on the five genes [Fig. 4D]. An ANN diagnostic model was developed based on the expression of five genes (Fig. 4D) and ROC analysis was conducted by using the data of the GSE142025 dataset to test accurate diagnostic value.The AUC was 0.996 for DUSP1, 0.992 for ZFP36, 0.971 for PDK4, 0.885 for CD44 and 0.761 for RGS4 (Fig. 4E).

### Identifcation of two ferroptosis regulation patterns facilitated by 16 FRGs

**Fig. 5:**
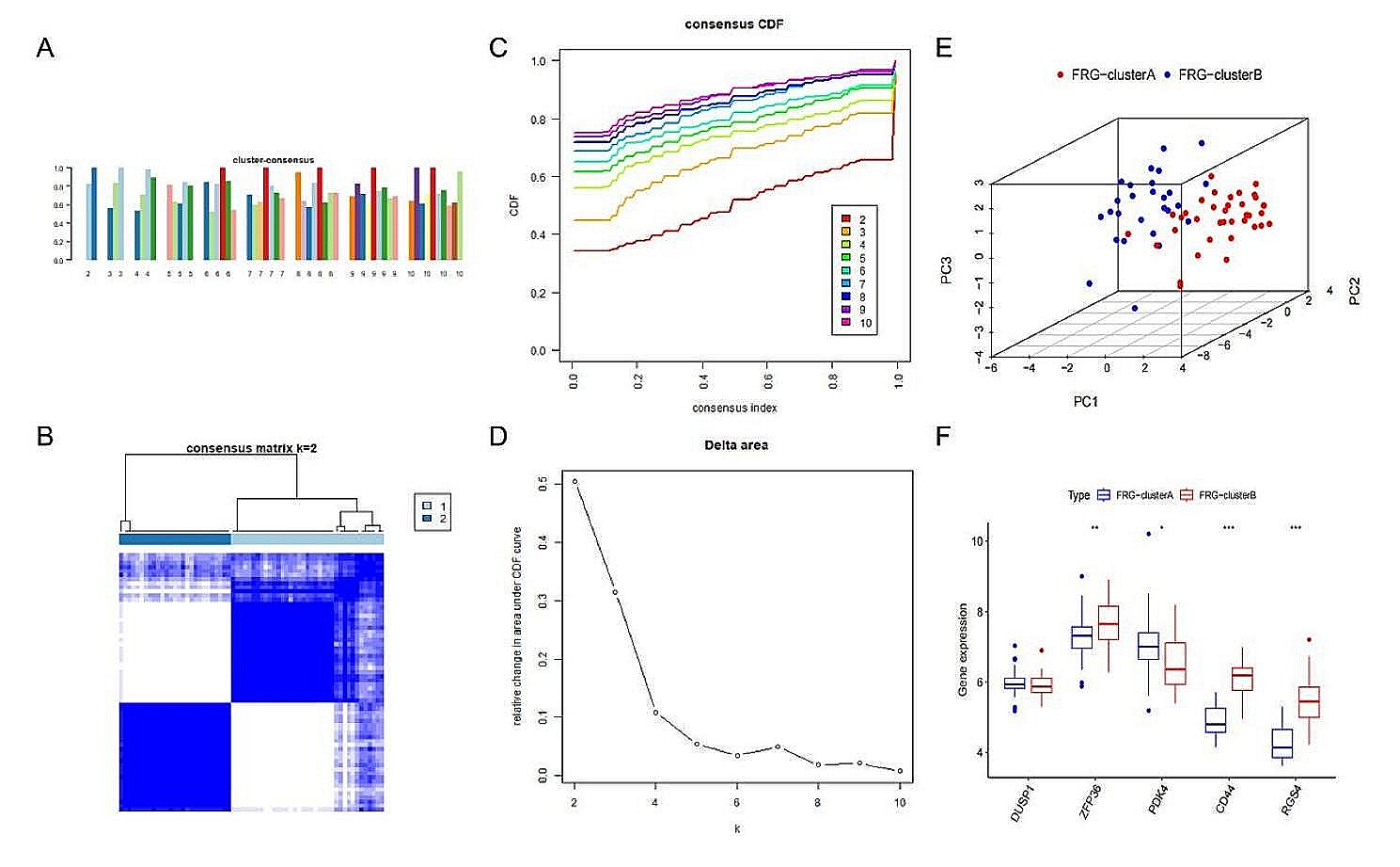
**(A-D)** Unsupervised clustering of 17 ferroptosis genes in a merged DKD cohort (*n* = 64, k = 2); **(E)** PCA analysis between two ferroptosis clusters;**(F)** The expression of five hub DE-FRGs in two ferroptosis clusters in merged cohort. *p < 0.05, **p < 0.01, ***p < 0.001

In order to further understand ferroptosis regulation patterns facilitated, we conducted consensus clustering analysis for subsequent study in DKD patients.Using cluster consensus, clustering heatmap and cumulative distribution function curves, we determined that the ideal value for k is 2 in subsequent analysis [Fig. 5A-D].The PCA analysis of two ferroptosis regulation patterns showed that the two ferroptosis clusters can be significantly distinguished and there are 37 DKD patients in cluster A and 27 DKD patients in cluster B [Fig. 5E].The expressions of five hub DE-FRGs all exhibit significant distinctions between two ferroptosis clusters except for DUSP1 [Fig. 5F].

### Immune cell Infiltration between CON and DKD,correlation analysis between immune infiltrating cells, key genes and different ferroptosis subtypes

**Fig. 6:**
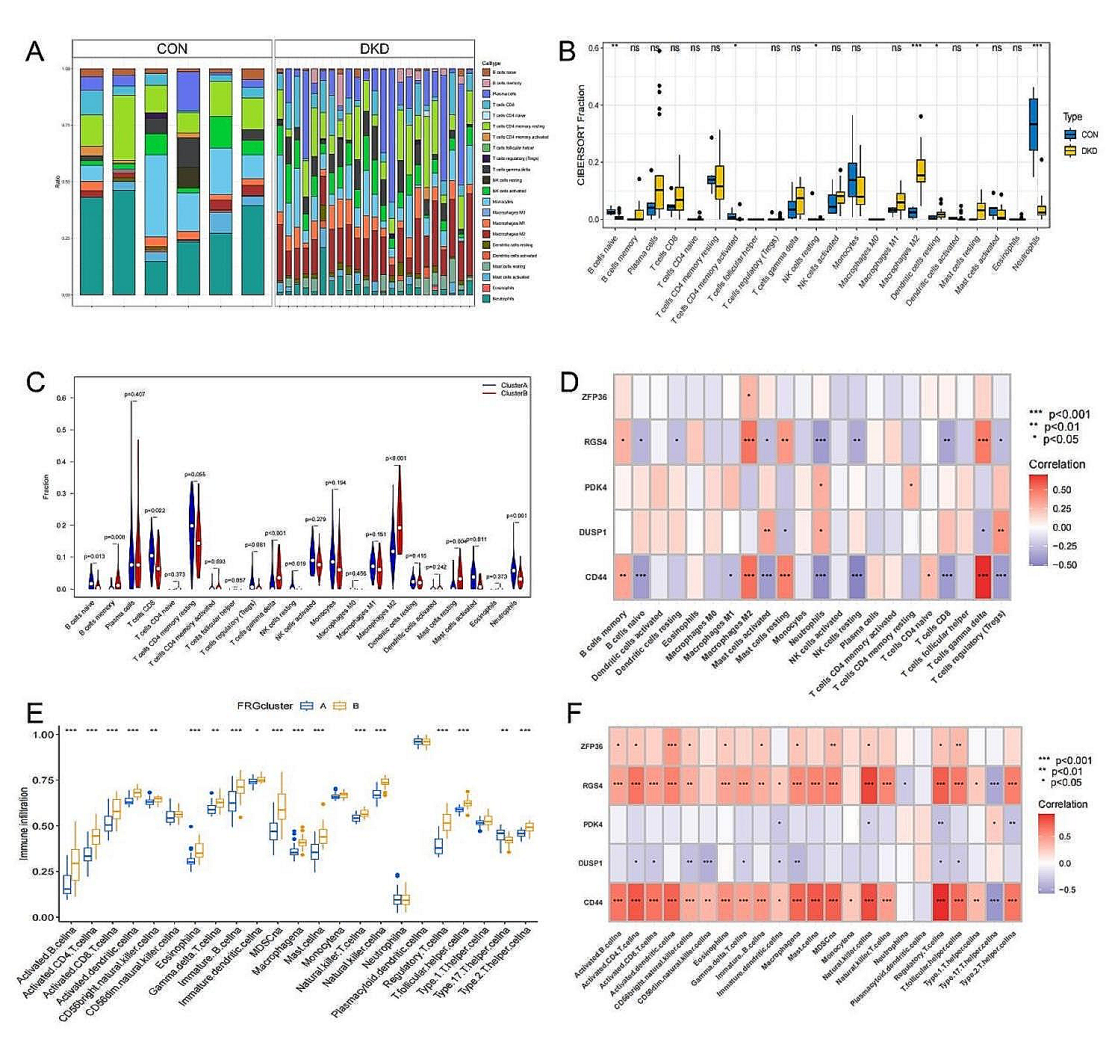
**(A)** The distribution of immune cells infiltration computed using the CIBERSORT algorithm; **(B)** The differentially expression of immune cells between CON and DKD; **(C)** Association between ferroptosis subtypes and immune infiltrating cells according to CIBERSORT algorithm; **(D)** Correlation between hub DE-FRGs and immune infiltrating cells based on CIBERSORT algorithm; **(E)** Association between ferroptosis subtypes and immune infiltrating cells according to ssGSEA algorithm; **(F)** Correlation between hub DE-FRGs and immune infiltrating cells based on ssGSEA algorithm. *p < 0.05, **p < 0.01, ***p < 0.001

Utilizing the CIBERSORT algorithm, we identified a pronounced variance in the composition of immune cells between DKD and CON [Fig. 6A].There were significantly differential expression of B cells naive, T cells CD4 memory activated, NK cells resting, Macrophages M2, Dendritic cells resting, Mast cells resting and Neutrophils between CON and DKD patients [Fig. 6B].Based on CIBERSORT algorithm,we found B cells naive, T cells CD8, NK cells resting, Mast cells activated and Neutrophils were significantly increased in Cluster A, while B cells memory, T cells gamma delta, Macrophages M2, Mast cells resting were significantly decreased (*p* < 0.05)[Fig. 6C].The correlation between hub DE-FRGs and immune cell infiltration levels from CIBERSORT methods in DKD were investigated using spearman analysis, from which we uncovered the subsequent significant connections: ZFP36 was associated with Macrophages M2, RGS4 with 11 immune cells and has the strongest correlation with Macrophages M2, Neutrophils and T cells gamma delta (*p* < 0.001),PDK4 with Neutrophils and T cells CD4 memory resting, DUSP1 with 5 immune cells, CD44 with 13 immune cells and has the strongest correlation with B cells naive, Macrophages M2 and T cells gamma delta etc. (*p* < 0.001) [Fig. 6D].Based on ssGSEA algorithm,we found 18 immune cells were significantly expressed between cluster A and cluster B (*p* < 0.05)[Fig. 6E].The correlation between hub DE-FRGs and immune cell infiltration levels from ssGSEA algorithm in DKD were also investigated using spearman analysis,ZFP36 was associated with 11 immune cells, RGS4 with 20 immune cells, PDK4 with 5 immune cells, DUSP1 with 9 immune cells, CD44 with 21 immune cells [Fig. 6F].

### The Immune microenvironment characteristics of two ferroptosis subtypes

**Fig. 7:**
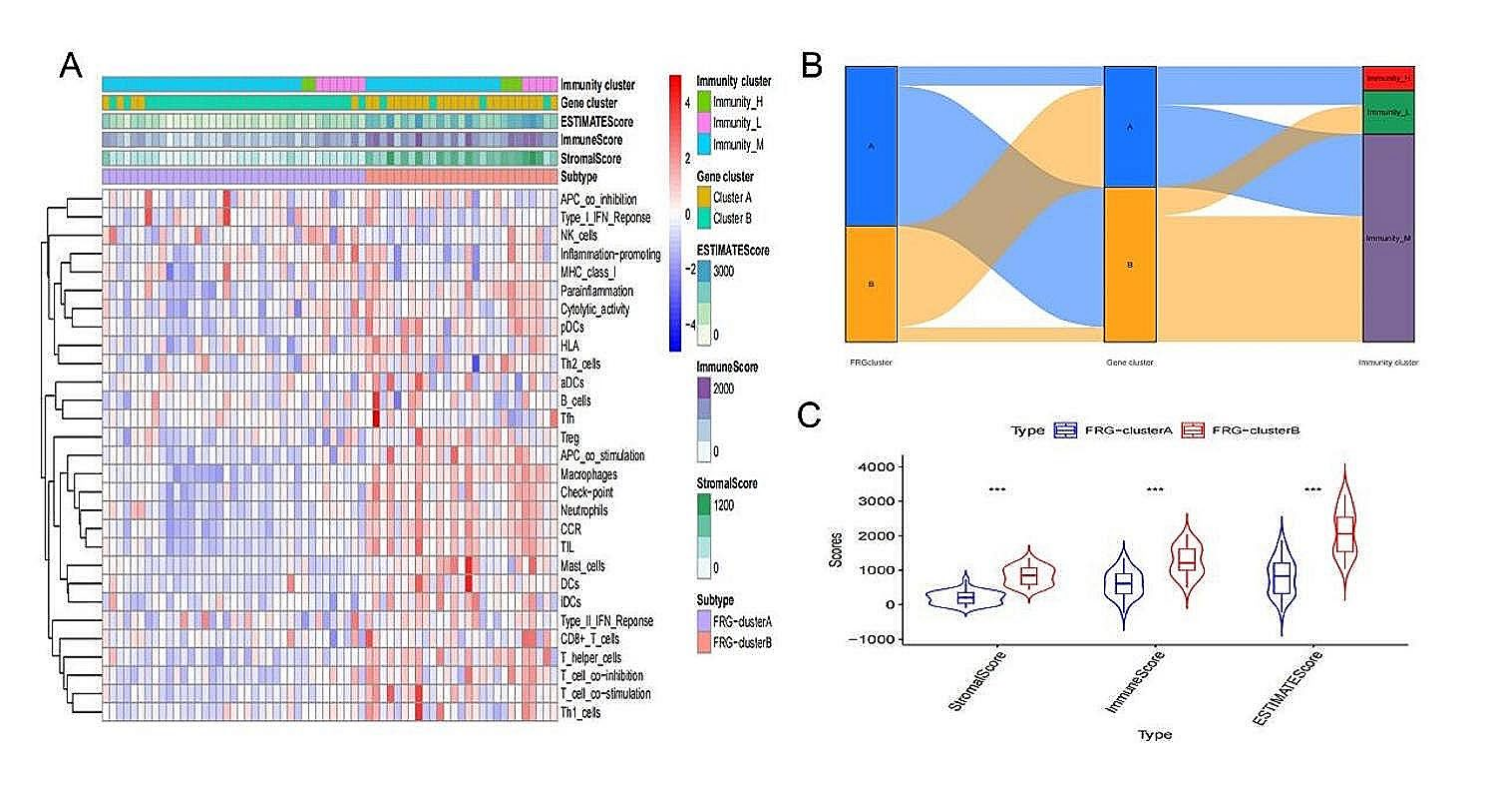
**(A)**The heatmap of the immune microenvironment between two ferroptosis clusters in DKD (FRG cluster A vs. cluster B); **(B)**The relationship of ferroptosis clusters,gene clusters and Immunity clusters; **(C)**The comparison of stromal, immune and ESTIMATE scores between FRG clusters.

We assessed the differences in the composition of the immune microenvironment, encompassing immune cells, stromal score, immune score, and ESTIMATE score across two ferroptosis subtypes using the estimate and ssGSEA algorithms [Fig. 7A]. The DKD samples were divided into two gene clusters (gene cluster A and B) using the consensus clustering method based on the expression of 270 DEGs. The DKD samples were further categorized into three immunity groups (Immunity High, Middle, and Low) according to the ssGSEA results. The FRG cluster, gene cluster, and immunity group were visualized using an alluvial diagram created with the ggalluvial R package [Fig. 7B]. In DKD patients, FRG cluster A demonstrated relatively lower stromal, immune, and ESTIMATE scores compared to FRG cluster B, and this difference is statistically significant (*p* < 0.001) [Fig. 7C].

### GSVA pathway analysis of ferroptosis subtypes

**Fig. 8:**
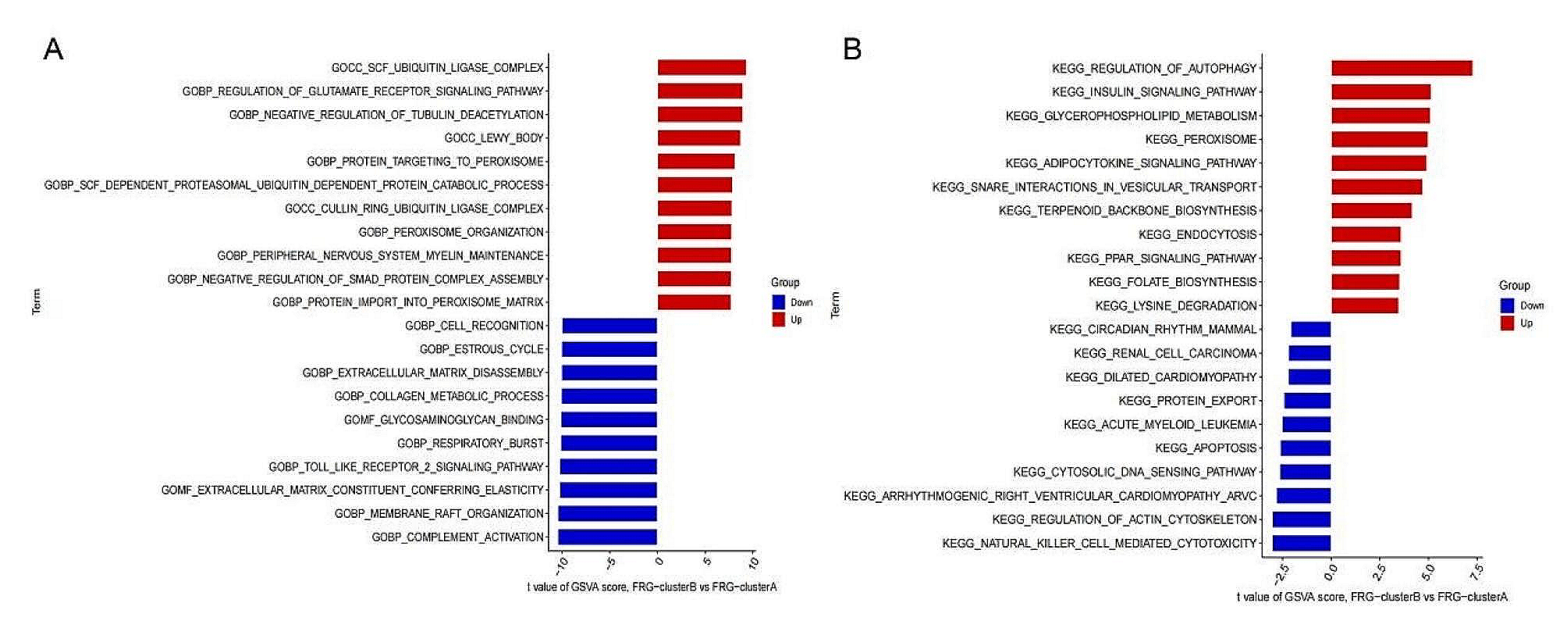
Differential pathway enrichment between FRG cluster A and cluster B based on **(A)** GO and **(B)** KEGG gene sets.

The GSVA pathway analysis unveiled a multitude of pathways significantly enriched based on the GO and KEGG gene set documents exhibited significant differences between two ferroptosis clusters.For instance,SCF UBIQUITIN LIGASE COMPLEX, CELL REGULATION and COMPLEMENT_ACTIVATION etc. (Fig. 8A), REGULATION OF AUTOPHAGY, PEROXISOME,CIRCADIAN RHYTHM MAMMAL,APOPTOSIS and other pathways (Fig. 8B).

### IHC staining results of kidney tissues

**Fig. 9:**
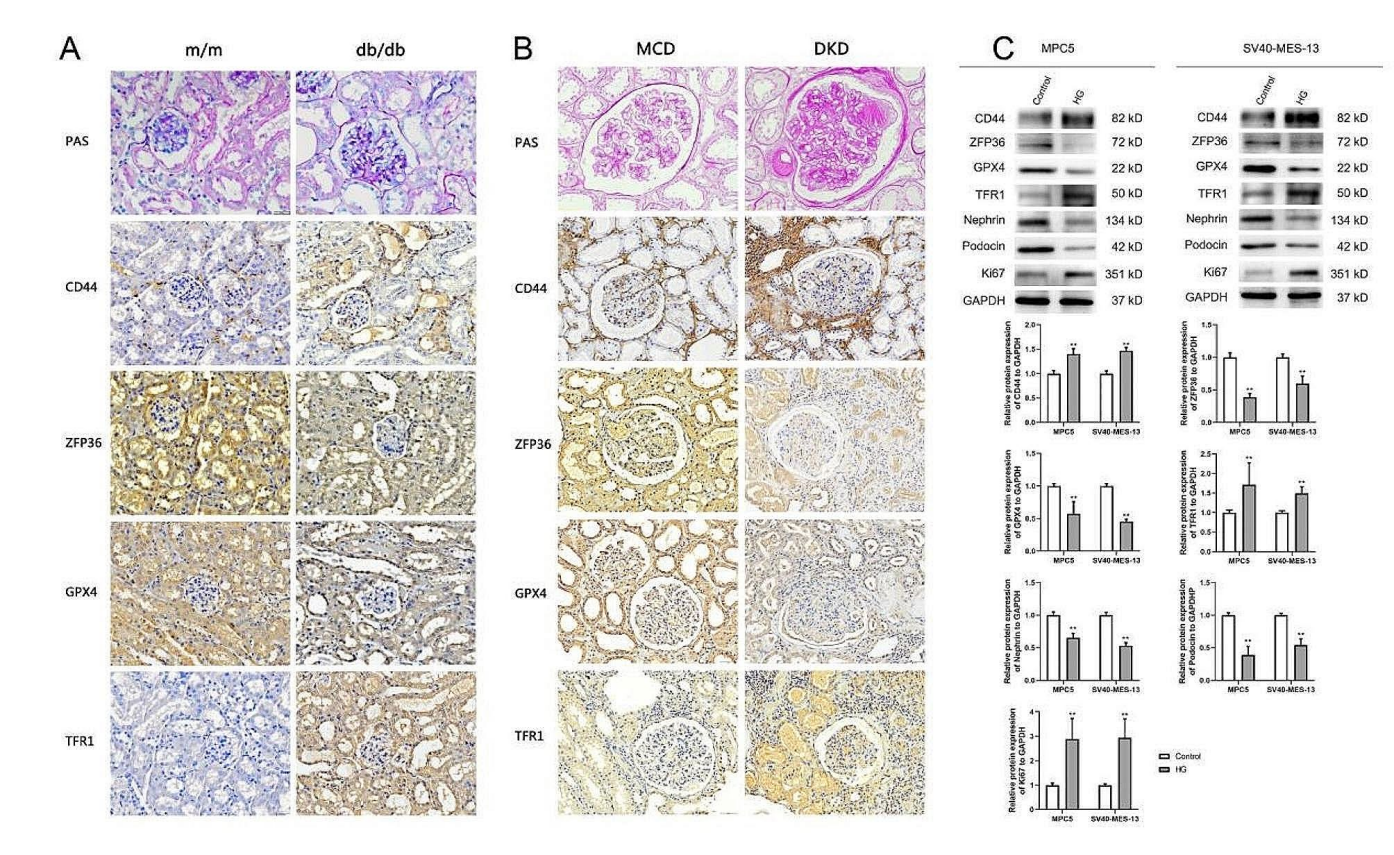
PAS staining and IHC staining results of kidney tissues of **(A)** experimental mice and **(B)** clinical patients;**(C)**Western Blotting for protein expression in MPC cells and SV40-MES-13 cells. * *p* < 0.05, * * *p* < 0.01

Compared with m/m mice, there were irregular nodular PAS positive material deposits in the glomeruli of db/db mice, glomerular mesangial matrix hyperplasia, and basement membrane thickening. In addition, IHC results showed that compared with m/m mice, the expression of CD44 and TFR1 in db/db mice was significantly increased, and the expression of ZFP36 and GPX4 was significantly reduced (Fig. 9A).Similar findings were also observed in renal tissue from DKD patients compared with MCD patients(Fig. 9B).

### Western blotting results of cells

Western Blotting showed that in MPC5 cells and SV40-MES-13 cells, the expression of CD44,TFR1 and Ki67 in HG group was significantly higher than that in Control group (*p* < 0.01), while the expression of ZFP36, GPX4, Nephrin and Podocin was significantly lower ( *p* < 0.01)(Fig. 9C).

## Discussion

Ferroptosis, a form of programmed cell death first proposed by Dixon et al. in 2012 [6], has been found to be involved in numerous pathological processes. Studies have shown that ferroptosis plays a crucial role in various cancer diseases, and an increasing amount of research is being conducted to investigate the relationship between ferroptosis and non-cancer diseases [7].DKD as the leading cause of chronic kidney disease worldwide, has also garnered significant interest in its connection with ferroptosis.Several studies have highlighted the significant role of ferroptosis in the initiation and advancement of DKD, resulting in renal tubular injury [11, 32], glomerular injury [13, 33] and directing interventions towards ferroptosis can mitigate interstitial inflammation and renal fibrosis [34].Furthermore, ferroptosis is often associated with an inflammatory response. To date, it has been identified to be involved in regulating the IME in a variety of diseases [35].Ferroptosis can modulate metabolic changes or the secretion of related substances between microbes and host cells or among host cells under inflammatory microenvironments. Furthermore, ferroptotic cells can recruit immune cells by releasing damage-associated molecular patterns, thereby inducing the generation of inflammatory microenvironments [35]. Nevertheless, there is limited understanding regarding the potential role of ferroptosis in shaping the IME in DKD. Therefore, it is urgent to fully elucidate the association between ferroptosis status with IME in DKD, which might offer a fresh avenue for exploring the fundamental molecular mechanisms.

In this study, we systematically examined the differential expression of FRGs in DKD and control kidney tissues by consolidating data from multiple databases and 16 DE-FRGs were finaly obtained. Functional annotation analysis confirmed that these genes are closely related to ferroptosis. Subsequently, according to the 16 DE-FRGs, two FRG clusters (cluster A and cluster B ) were established by by consensus clustering, and was correlated with IME characteristics, including immune cells, stromal score, immune score and ESTIMATE score. Five ferroptosis genes (DUSP1,ZFP36,PDK4,CD44 and RGS4) were closely associated with DKD by machine learning algorithms.The diagnostic model based on the five ferroptosis genes had a good diagnosis performance in distinguishing DKD from control patients in external validation.The expression of DUSP1 between two FRG clusters were of no sense,the other four genes were with significance.ZFP36,CD44 and PDK4 has a better performance in diagnosing DKD with AUC > 0.85.Moreover, ZFP 36 and CD44 has a strong correlation with multiple immune cells infiltration means they may play a vital role between IME and ferroptosis. Finally, a plausible identification revealed the upregulation of the ferroptosis regulator ZFP36 and GPX4 and the downregulation of the ferroptosis regulator CD44 and TRF1 in DKD through the utilization of IHC in DKD tissues and db/db spontaneous type 2 diabetes mouse model and WB in mouse podocyte MPC5 and mesangial SV40-MES-13 cells under HG conditions.

For a more comprehensive exploration of the crosstalk between Ferroptosis subtypes and IME, as well as the interaction between key FRGs and immune cells in DKD, we employed a variety of immune assessment algorithms, including CIBERSORT, ESTIMATE and ssGSEA.Based on CIBERSORT algorithm, we observed that the infiltration of B cells naive, T cells CD4 memory activated, NK cells resting, Macrophages M2, Dendritic cells resting, Mast cells resting and Neutrophils were the significantly different between CON and DKD,which is mostly consistent with Zhang,XQ et al’s findings [36].Another notable observation is the increased presence of both M1 and M2 macrophages in DKD patients, with the elevation of M2 macrophages showing the most pronounced difference.Investigations have unveiled that M1 macrophages are linked to the initial phases of DKD damage [37], exhibiting an M2 phenotype during the DKD’s reparative stage [38] and it could prevent podocyte damage by inhibiting M1 macrophage activation and promoting M2 macrophage transformation [39].Notably, our research found that M2 macrophages were significantly increased in DKD may be due to M1 macrophages can ultimately transform into M2 macrophages.However,the role of M2 macrophages in the fibrosis of DKD is controversial, since they can participant in anti-inflammatory by secreting a number of anti-inflammatory molecules, for example IL-10,as well as promotes kidney fibrosis at the advanced stage of DKD by secreting a list of proinflammatory mediators,such as IL-1β,transforming growth factor-beta (TGF-β) and chemotactic protein-1 (MCP-1) [40].Furthermore, the reduction in one category of macrophages can trigger a transformation to another type of macrophage in response to the adverse conditions within the diseased microenvironment. Macrophages play a pivotal role in maintaining tissue balance by overseeing inflammation and controlling iron, lipid, and amino acid metabolism through their unique abilities, such as phagocytosis and efferocytosis, as well as the secretion of cytokines and the generation of reactive oxygen species (ROS) under varying polarization conditions since ferroptosis is often associated with concurrent inflammatory responses [41],it’s clear that there is a connection between macrophages and ferroptosis.

According to the immune cells infiltration results from the three immune estimation algorithms, multiple immune cells were significant difference between FRG-cluster A and FRG-Cluster B. Among them, macrophages are the most important. In addition, two crucial ferroptosis genes, CD44 and ZFP36, experimentally validated, exhibited a positive correlation with macrophages, with CD44 showing a particularly strong association. ZFP36, namely zinc finger protein 36, exerts an influence on the stability of TNF-α mRNA and a recent study has revealed that the disruption of ZFP36 in mice led to the development of a multifaceted inflammatory syndrome due to elevated TNF-α production [42] and further attracts macrophage infiltration. In this study, we first revealed ZFP36 protein expression were downregulated in DKD kidney tissues and in podocyte MPC5 and mesangial SV40-MES-13 cells after HG stimulation,while the downregulation of ZFP36 can trigger the activation of ferritinophagy and the initiation of ferroptosis [43].CD44, a cell-surface glycoprotein, has been extensively researched, with its functions encompassing a wide range of physiological and pathological activities, such as cell proliferation, adhesion, migration, angiogenesis, inflammation, and cytoskeleton rearrangement [44]. Nevertheless, recent findings also shed light on CD44’s involvement in metabolism, particularly its role in insulin resistance associated with obesity and diabetes and plays a critical role in maintaining glucose and lipid homeostasis [44].Interestingly, targeted inhibition of CD44/SLC7A11 interactions could render tumor cells more susceptible to ferroptosis [45].To the best of our understanding, our team is the first to unveil that CD44 protein expression is upregulated in diabetic kidney disease (DKD) kidney tissues, as well as in podocyte and mesangial cells exposed to high glucose (HG) stimulation. Moreover, CD44 influences epigenetic adaptability by controlling the process of iron endocytosis [46] and the expression of CD44 in renal tissue is associated with an increase in complement levels in urine and renal fibrosis [47].Our research indicated that CD44 and ZFP36 maybe a potential regulator between IME and ferroptosis in DKD.Notably, there was a pronounced difference in IME between FRG-cluster A and FRG-Cluster B, suggesting that patients in FRG-Cluster B had a heightened capacity for immune evasion and this may be due to autophagy, apoptosis or complement activation participate in the regulation of ferroptosis phenotypes according to GSVA analysis.

This study still has certain limitations that we aim to address progressively in the future.Firstly, Despite our pioneering discovery and validation of the functions of ferroptosis regulators CD44 and ZFP36 in DKD and immune infiltration, it remains imperative to ascertain the potential biological roles of ZFP36 and CD44.Another limitation is the absence of verification for the interactions between CD44, ZFP36, ferroptosis, and macrophages in DKD through other functional or in vitro studies. This will be a primary area of focus for our future research.

To summarize, this study identified two molecular FRG subtypes in DKD using consensus clustering and immune analyses indicate that the dysregulation of the IME may be responsible for triggering ferroptosis. Additionally, we have identified and confirmed that CD44 and ZFP36 represent two promising biomarkers and have a positive correlation with macrophages, offering potential efficacy in the recognition and development of mRNA vaccines for individuals with DKD.

### Electronic supplementary material

Below is the link to the electronic supplementary material.

Supplementary Material 1

## Data Availability

All datasets and materials utilized in this research are incorporated within the paper and can be obtained from the corresponding author upon a reasonable request.

## References

[1] GBD Chronic Kidney Disease Collaboration (2020). Global, regional, and national burden of chronic kidney disease, 1990–2017: a systematic analysis for the global burden of Disease Study 2017. Lancet.

[2] International Diabetes Federation.IDF Diabetes Atlas-. 10th Edition. Available at http://www.diabetesatlas.org.

[3] Kidney Disease: Improving Global Outcomes (KDIGO) Diabetes Work Group (2022). KDIGO 2022 Clinical Practice Guideline for Diabetes Management in chronic kidney disease. Kidney Int.

[4] Bayır H, Dixon SJ, Tyurina YY, Kellum JA, Kagan VE (2023). Ferroptotic mechanisms and therapeutic targeting of iron metabolism and lipid peroxidation in the kidney. Nat Rev Nephrol.

[5] Zhou Y, Zhang J, Guan Q, Tao X, Wang J, Li W (2022). The role of ferroptosis in the development of acute and chronic kidney diseases. J Cell Physiol.

[6] Dixon SJ, Lemberg KM, Lamprecht MR (2012). Ferroptosis: an iron-dependent form of nonapoptotic cell death. Cell.

[7] Jiang X, Stockwell BR, Conrad M (2021). Ferroptosis: mechanisms, biology and role in disease. Nat Rev Mol Cell Biol.

[8] Stockwell BR (2022). Ferroptosis turns 10: emerging mechanisms, physiological functions, and therapeutic applications. Cell.

[9] Lei G, Zhuang L, Gan B (2022). Targeting ferroptosis as a vulnerability in cancer. Nat Rev Cancer.

[10] Kim R, Hashimoto A, Markosyan N (2022). Ferroptosis of tumour neutrophils causes immune suppression in cancer. Nature.

[11] Wang Y, Bi R, Quan F (2020). Ferroptosis involves in renal tubular cell death in diabetic nephropathy. Eur J Pharmacol.

[12] Li S, Zheng L, Zhang J, Liu X, Wu Z (2021). Inhibition of ferroptosis by up-regulating Nrf2 delayed the progression of diabetic nephropathy. Free Radic Biol Med.

[13] Zhang Q, Hu Y, Hu JE (2021). Sp1-mediated upregulation of Prdx6 expression prevents podocyte injury in diabetic nephropathy via mitigation of oxidative stress and ferroptosis. Life Sci.

[14] Chen X, Kang R, Kroemer G, Tang D (2021). Broadening horizons: the role of ferroptosis in cancer. Nat Rev Clin Oncol.

[15] Chen X, Kang R, Kroemer G, Tang D (2021). Ferroptosis in infection, inflammation, and immunity. J Exp Med.

[16] Hsu SK, Li CY, Lin IL (2021). Inflammation-related pyroptosis, a novel programmed cell death pathway, and its crosstalk with immune therapy in cancer treatment. Theranostics.

[17] Woroniecka KI, Park AS, Mohtat D, Thomas DB, Pullman JM, Susztak K (2011). Transcriptome analysis of human diabetic kidney disease. Diabetes.

[18] Grayson PC, Eddy S, Taroni JN, Lightfoot YL, Mariani L, Parikh H, Lindenmeyer MT (2018). Metabolic pathways and immunometabolism in rare kidney diseases. Ann Rheum Dis.

[19] Pan Y, Jiang S, Hou Q (2018). Dissection of glomerular Transcriptional Profile in patients with Diabetic Nephropathy: SRGAP2a protects Podocyte structure and function. Diabetes.

[20] Ju W, Greene CS, Eichinger F (2013). Defining cell-type specificity at the transcriptional level in human disease. Genome Res.

[21] Fan Y, Yi Z, D’Agati VD (2019). Comparison of kidney transcriptomic profiles of early and Advanced Diabetic Nephropathy reveals potential New mechanisms for Disease Progression. Diabetes.

[22] Zhou N, Yuan X, Du Q, Zhang Z, Shi X, Bao J, Ning Y, Peng L (2023). FerrDb V2: update of the manually curated database of ferroptosis regulators and ferroptosis-disease associations. Nucleic Acids Res.

[23] Lindsey JK (1999). A review of some extensions to generalized linear models. Stat Med.

[24] Rigatti SJ (2017). Random Forest. J Insur Med.

[25] Tan M, Pu J, Zheng B (2014). Optimization of breast mass classification using sequential forward floating selection (SFFS) and a support vector machine (SVM) model. Int J Comput Assist Radiol Surg.

[26] Chen T, Guestrin C, XGBoost. A scalable tree boosting system. Proceedings of the 22nd ACM SIGKDD International Conference on Knowledge Discovery and Data Mining; August 13–17, 2016, San Francisco, California. ACM, 2016; pp.785–94.

[27] Timmerman ME, Ceulemans E, De Roover K, Van Leeuwen K (2013). Subspace K-means clustering. Behav Res Methods.

[28] Wilkerson MD, Hayes DN (2010). ConsensusClusterPlus: a class discovery tool with confidence assessments and item tracking. Bioinformatics.

[29] Newman AM, Liu CL, Green MR (2015). Robust enumeration of cell subsets from tissue expression profiles. Nat Methods.

[30] Jia Q, Wu W, Wang Y (2018). Local mutational diversity drives intratumoral immune heterogeneity in non-small cell lung cancer. Nat Commun.

[31] He Y, Jiang Z, Chen C, Wang X (2018). Classification of triple-negative breast cancers based on immunogenomic profiling. J Exp Clin Cancer Res.

[32] Kim S, Kang SW, Joo J (2021). Characterization of ferroptosis in kidney tubular cell death under diabetic conditions. Cell Death Dis.

[33] Wu Y, Zhao Y, Yang HZ, Wang YJ, Chen Y (2021). HMGB1 regulates ferroptosis through Nrf2 pathway in mesangial cells in response to high glucose. Biosci Rep.

[34] Zhou L, Xue X, Hou Q, Dai C (2021). Targeting ferroptosis attenuates interstitial inflammation and kidney fibrosis. Kidney Dis (Basel).

[35] Dou J, Liu X, Yang L, Huang D, Tan X (2022). Ferroptosis interaction with inflammatory microenvironments: mechanism, biology, and treatment. Biomed Pharmacother.

[36] Zhang X, Chao P, Zhang L (2023). Single-cell RNA and transcriptome sequencing profiles identify immune-associated key genes in the development of diabetic kidney disease. Front Immunol.

[37] Fu J, Sun Z, Wang X (2022). The single-cell landscape of kidney immune cells reveals transcriptional heterogeneity in early diabetic kidney disease. Kidney Int.

[38] Calle P, Hotter G (2020). Macrophage phenotype and fibrosis in Diabetic Nephropathy. Int J Mol Sci.

[39] Jiandong L, Yang Y, Peng J (2019). Trichosanthes kirilowii lectin ameliorates streptozocin-induced kidney injury via modulation of the balance between M1/M2 phenotype macrophage. Biomed Pharmacother.

[40] Yan J, Li X, Liu N, He JC, Zhong Y (2023). Relationship between macrophages and tissue microenvironments in Diabetic kidneys. Biomedicines.

[41] Yang Y, Wang Y, Guo L, Gao W, Tang TL, Yan M (2022). Interaction between macrophages and ferroptosis. Cell Death Dis.

[42] Zhang Y, Li NF, Abulikemu S (2015). Relationship between zinc finger protein 36 (ZFP36) gene polymorphisms and obstructive sleep apnea. Genet Mol Res.

[43] Zhang Z, Guo M, Li Y (2020). RNA-binding protein ZFP36/TTP protects against ferroptosis by regulating autophagy signaling pathway in hepatic stellate cells. Autophagy.

[44] Weng X, Maxwell-Warburton S, Hasib A, Ma L, Kang L (2022). The membrane receptor CD44: novel insights into metabolism. Trends Endocrinol Metab.

[45] Bian Z, Sun X, Liu L (2023). Sodium Butyrate induces CRC Cell Ferroptosis via the CD44/SLC7A11 pathway and exhibits a synergistic therapeutic effect with Erastin. Cancers (Basel).

[46] Müller S, Sindikubwabo F, Cañeque T (2020). CD44 regulates epigenetic plasticity by mediating iron endocytosis. Nat Chem.

[47] Chebotareva N, Vinogradov A, Tsoy L (2023). CD44 expression in renal tissue is Associated with an increase in urinary levels of Complement Components in Chronic glomerulopathies. Int J Mol Sci.

