# Supplementary material for: Unraveling the interplay of ferroptosis and immune dysregulation in diabetic kidney disease: a comprehensive molecular analysis
Source: Diabetol Metab Syndr. 2024 Apr 20;16:86. doi: 10.1186/s13098-024-01316-w (PMC11032000; doi:10.1186/s13098-024-01316-w)
Supplement: Supplementary file 1 — Supplementary Material 1 [file 13098_2024_1316_MOESM1_ESM.docx]

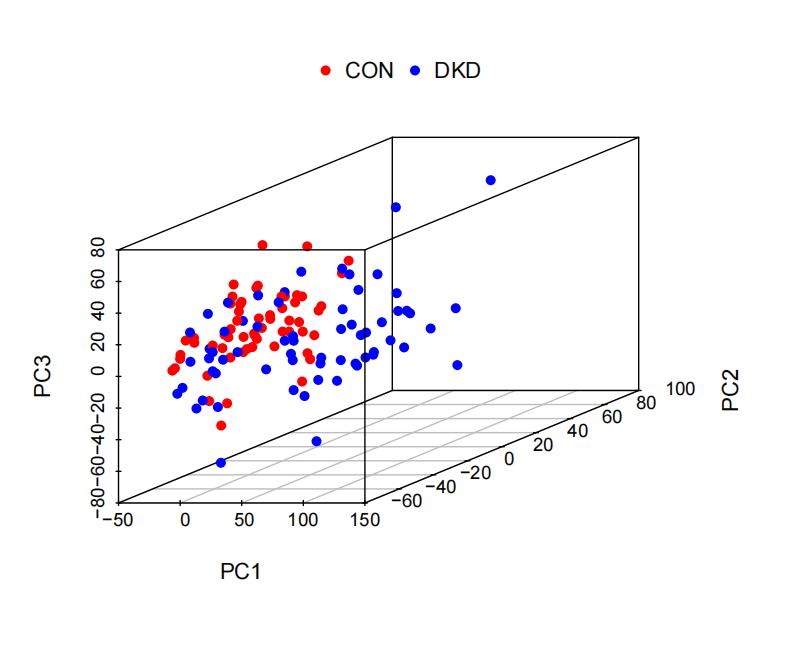


**Fig.S1.**PCA analysis between CON and DKD samples.

PCA:Principal Component Analysis ; CON:Control; DKD：Diabetic kidney disease.


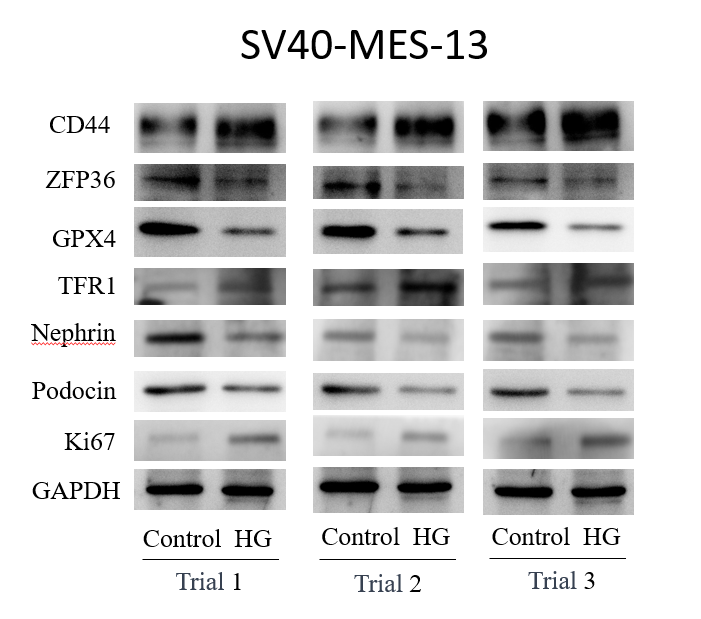


**Fig.S2.**Western Blotting for protein expression in SV40-MES-13 cells repeated three times.


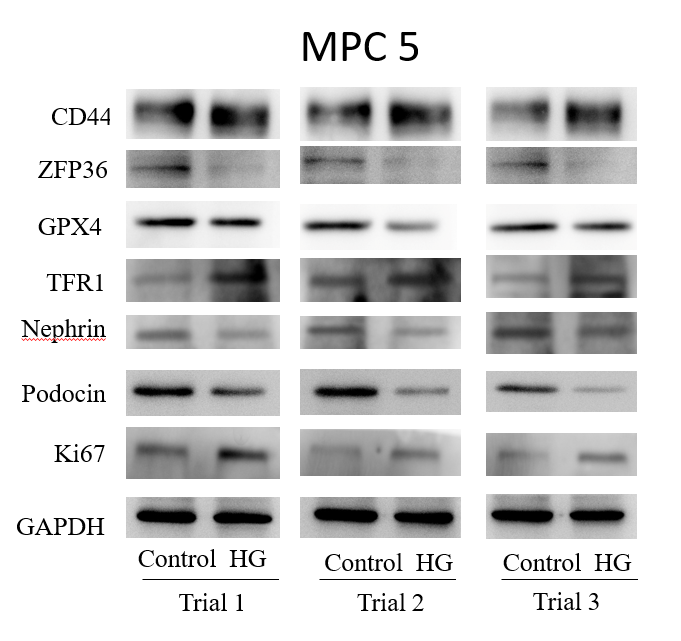


**Fig.S3.**Western Blotting for protein expression in MPC cells repeated three times.
